# Education Research: A Qualitative Analysis of the Role of Social Media in Neurology Trainees' Professional Identity Formation

**DOI:** 10.1212/NE9.0000000000200307

**Published:** 2026-04-22

**Authors:** Gabriela Figueiredo Pucci, Galina Gheihman, Catherine S.W. Albin

**Affiliations:** 1 Department of Neurology, University of Pittsburgh Medical Center, Pittsburgh, PA;; 2 Department of Neurology, Mass General Brigham and Harvard Medical School, Boston, MA; and; 3 Division of Neurocritical Care, Department of Neurology, Emory University School of Medicine, Atlanta, GA.

## Abstract

**Background and Objectives:**

Professional Identity Formation (PIF) is a dynamic process by which students and trainees integrate the professional values, expectations, and community norms of the medical community into their personal identity. PIF is often understood as occurring through socialization into a Community of Practice (CoP). Despite the growing use of social media by medical trainees, little is known about how PIF unfolds in virtual environments and the extent to which social media use can influence the career development of trainees. In this study, we explored how participation in a social media community (“#NeuroTwitter”) influenced PIF and career development among neurology trainees.

**Methods:**

Informed by CoP theory, we developed a semistructured interview guide. We used purposive criterion sampling and snowball sampling to identify and recruit neurology trainee “superusers” on X, the social media platform formerly known as Twitter. Eligible participants were invited to complete a one-on-one virtual semistructured interview between August and November 2024. We used inductive thematic analysis to identify key themes and interpreted these through the lens of PIF.

**Results:**

We interviewed 18 X superusers (13 residents, 3 fellows, and 2 first-year attendings) from 17 institutions in the United States. We identified 4 key themes of how the #NeuroTwitter community influenced the trainees' PIF: (1) Access to Neurology, (2) #NeuroTwitter as a CoP, (3) Translation of #NeuroTwitter involvement into “in real-life” achievements, and (4) Challenges in navigating the digital environment. Many neurology trainees shared how the #NeuroTwitter Community was equitable, nonhierarchal, and generous and described how involvement accelerated professional opportunities. However, accruing these benefits required navigating the platform and tensions between their professional self, digital self, and “real” self.

**Discussion:**

#NeuroTwitter functioned as a CoP which fostered a sense of belonging and accelerated PIF among deliberate superusers. However, participants also described identity friction and platform concerns. Although PIF that happens in virtual communities is important, it should be viewed as complementary—rather than a replacement—to PIF occurring in real-life.

## Introduction

Professional identity is the image an individual has of oneself, based on their professional roles within a professional field.^1^ Professional identity formation (PIF) is the complex socialization process that occurs throughout medical training to ensure a trainee learns to “think, act, and feel like a physician” and represents the sum of influences impacting 3 domains: individual, relational, and collective.^2,3^ A central aim of medical education is to help learners adapt and eventually internalize the professional identity of a physician.^4^

Socialization is critical for PIF within the medical profession; socialization in this context is the process by which an individual moves from peripheral participation to full participation in a group or process.^5^ This transformation occurs within the community one seeks to join and is often described using Community of Practice (CoP) theory.^6^ In a CoP, individuals with shared interests (the Community) and a unifying area of focus (the Domain) collectively build and refine the group's core knowledge through sustained interaction (the Practice).^7,8^ For physicians, professional identity has traditionally been developed as part of the medical school curriculum, both formal and “hidden,” and within the physical spaces of the medical school, hospital, and clinic.^6,9-11^ However, many of today's trainees are members of “Generation Z” or young “Millennials” who have grown up with technology present in all aspects of their lives. Since birth, social media has allowed them to inhabit both physical and virtual spaces.^12^ Consequently, there has been a growing recognition that PIF among trainees may be influenced by digital networks as well as in-person networks and that digital identity formation may parallel, contribute to, and blur with PIF.^13,14^

The exploration of digital identity formation is particularly important to consider on social media platforms as they are becoming more widely used in the medical community. The #intEHRAct survey demonstrated healthcare professionals have gravitated to social media to stay up to date on recent literature, network, and review rare or interesting cases.^15^ A cross-sectional survey revealed over 85% of healthcare practitioners agreed social media could be an effective tool for education.^16^ Within the neurology community, a 2023 multisite cross-sectional survey demonstrated that 70% of participants used social media professionally, with X (formally Twitter) and Facebook being preferred.^17^

Once on a social media platform, hashtags enable users to tag content for visibility to other users with shared interests. #NeuroTwitter is one such hashtag, situated within the broader scope of #MedTwitter, which enables geographically dispersed users to become collectively unified by a shared interest in neurology.

The role being part of a virtual community may play in neurology trainees' PIF has been underexplored. Using CoP and PIF as guiding frameworks, we asked how deliberate engagement on the social media platform X might influence trainees' PIF. We assumed an interpretivist approach, holding there is no single, objective truth, but rather that many subjective perspectives can help explain a socially constructed reality. In this descriptive qualitative study, we identified and interviewed high utilizers of X (which we termed “superusers”) among neurology trainees. Through this work, we aim to explore how purposeful use of social media affected PIF in neurology trainees. By better understanding how trainees engage with digital communities, we hope our results elucidate how these communities support PIF, what tensions arise, and ultimately equip educators with a better understanding of how they can promote integration of PIF that occurs in both virtual and clinical spaces.

## Methods

### Recruitment and Sampling

We defined X “superusers” as having >400 followers and having posted at least once in the prior month at the time of study enrollment. As there was no precedent for what defined a social media superuser, we estimated that having >400 followers demonstrated a user had more followers digitally than in their personal “in real-life” social network. Initial participants were selected purposively from X based on the research team member's knowledge of highly involved individuals. To recruit a diverse sample meeting the superuser criterion, a subsequent snowball approach was employed (interviewees recommended additional participants). Participants were recruited via email or X. All interviews were conducted between September and November 2024.

### Guide Development and Semistructured Interviews

We developed a semistructured interview guide informed by CoP theory and prior research exploring PIF.^18^ The guide was flexible, and interviewers were trained to probe to obtain specific examples and further elaboration. This guide was reviewed by an expert in medical education and qualitative research (Supplement 1 for full guide).

The authors conducted 30–45-minute semistructured interviews virtually (Zoom or Microsoft Teams). Participants were informed of the purpose of the study and the background and experiences of the researchers, though not their specific identities. Interviews were transcribed using Rev.com and Microsoft Teams transcription, reviewed by each interviewer for accuracy, and deidentified. Data collection was completed before data analysis commenced.

### Data Analysis

Qualitative inductive thematic analysis was completed by the 3 study investigators using the coding software Dedoose (SocioCultural Research Consultants, LLC, Los Angeles, CA). All transcripts were independently read. An initial codebook was developed and then revised and finalized through investigator meetings. Next, all team members coded 2 transcripts. Agreement and mutual understanding of the codebook was achieved. The remaining transcripts were divided up and coded by 2 coders. Coded transcripts were reviewed by all team members, and discrepancies were discussed and consolidated. We reached saturation as no new codes were identified as we completed coding.^19^ All authors reviewed and approved the final codes.^20^ Themes derived from the coding process were proposed and revised, interpreted through the lens of CoP and PIF. While themes were inductively derived, the domains reported reflect larger categorizations of themes and were organized after coding.

We sought to promote the trustworthiness of our analysis through several strategies.^19^ For transferability, we offer a description of the training background and stage of training for each participant. For credibility, we used in-depth engagement with the interview transcripts, double coding, and reached sufficient information power.^21^ We prepared to conduct additional interviews if we did not reach saturation; however, when completing the coding of our initial set of interviews we found no new codes were identified. Information on the researcher's backgrounds is included in eMethods 1. Given that participants might be aware of the prominence of their interviewer or other authors in the neurology digital community and this could shape their responses to our questions, we paired individuals without a prior in-person relationship for interviews and did not reveal the identities of other team members. We maintained awareness of how this might influence the openness of our participants' responses during data analysis. Confirmability was seen to through maintaining an audit trail documenting analysis decisions.

### Ethics Approval

This study was approved by the Emory University Institutional Review Board (#00005804). Verbal informed consent was obtained from participants before their involvement.

### Data Availability

The data from this study are available on request from the corresponding author.

## Results

### Participant Demographics

Fourteen X superusers were invited to participate. From those, 12 consented and were enrolled. During the interviews, 6 more individuals were identified using snowball sampling, and also consented, for a total of 18 participants. Participants represented 17 unique institutions. Eleven (61.11%) were IMGs and 7 (38.89%) USMGs. Participants included 6 (33.33%) PGY1s, 1 (5.55%) PGY2, 3 (16.67%) PGY3s, 3 (16.67%) PGY4s, 3 (16.67%) fellows, and 2 (11.11%) attendings (Table 1).

**Table 1:** Participant Demographics

Level of training	Medical graduate type	Number of followers on X
PGY1	USMG	461
PGY1	IMG	579
PGY1	USMG	716
PGY1	IMG	972
PGY1	IMG	1,193
PGY1	IMG	3,734
PGY2	USMG	1954
PGY3	IMG	823
PGY3	USMG	1766
PGY3	IMG	3,117
PGY4	IMG	1,372
PGY4	IMG	1,682
PGY4	IMG	1803
Fellow	IMG	621
Fellow	USMG	3,538
Fellow	USMG	3,819
Junior (1st y) Attending	USMG	1,327
Junior (1st y) Attending	IMG	2,673

Abbreviations: Level of training as of August 2024; PGY = post graduate year; IMG = International Medical Graduate; USMG = United States Medical Graduate; Number of followers on X as of August-November 2024 depending on when the participant was recruited.

### Four Major Domains

We identified 4 major domains regarding the role of #NeuroTwitter in PIF: (1) X offers access to the Neurology community, (2) #NeuroTwitter as a CoP, (3) Translation of #NeuroTwitter involvement into “in real-life” achievements, and (4) Challenges in navigating the digital environment. Each domain encompasses several major themes and subthemes, which are described below and summarized with illustrative quotations in Table 2, and in eTable 1, available in the Supplementary Materials.

**Table 2:** 4 Major Domains, Major Themes, and Subthemes Related to Professional Identity Formation in Digital Spaces, From a Qualitative Study of Neurology Trainees on #Neurotwitter

Domains and major themes	Sample quotations
Domain 1: Access to Neurology	
Reasons for joining	“So, the first thing that made me join was building connections. […] So that's how I initially got into Twitter and found great, amazing mentors.” (P5)
Beyond [their] institution	“My medical school actually didn't have a core clerkship, which is a lot of the reason why I ended up looking to social media to help build these connections and find mentors that weren't at my home institution.” (P7)
Opportunity for networking	“I think a lot of people who I've connected with on X initially had never even met them in person, but [it helped] then getting to meet them at conferences.” (P14)
Domain 2: #NeuroTwitter as a Community of Practice	
Community norms	“So, Twitter, it's been a great tool for reaching out and just there is no limit. There is no boundary. If you do it professionally and with respect, you can talk to anyone anywhere.” (P11)
Role models and mentorship	“I think that Twitter has democratized mentorship in the sense of many people have open direct messages on X. There are many people around the country—around the world!—who are eager and want to help.” (P15)
Belonging	“When you go on there and you find someone who really believes in something that you believe in and you get to exchange ideas and share that passion, I find that really, really joyful.” (P13)
Engagement	“I've been using [X] to reflect on milestones that I have in residency. Reflections that I've come up with, thoughts that I wish I knew a year ago to share with others.” (P7)
Domain 3: Translation of #NeuroTwitter involvement into “in real-life” achievements	
Acquisition of knowledge	“But then I realized that if I was on social media, and I had a network of physicians, of professionals, [who] would post that cool stuff, then I would know that that cool stuff exists, and I would read that.” (P3)
Virtual to real-life mentoring	“I cannot talk enough about how Twitter seriously changed probably the trajectory in my career, even though I'm in the very early stages of it. [The relationship with my mentor] is the best part that's come out of my Twitter experience.” (P7)
Reputation and visibility	“It was really helpful to get my name out there, and I was able to connect me to a larger global neurology audience, which was able to create name recognition for me going forward.” (P15)
Domain 4: Challenges of navigating the digital environment	
Tension between digital, personal, and professional identity	“One of the unseen side effects and downfalls of social media is if we aren't truly being authentic, it makes it more difficult to be vulnerable.” (P7)
Time management	“I do not love the fact that, you know, it's kind of addictive. So, you know, if you have it on your phone, you might […] be somewhere at work and looking at it, and I have had to distance myself for that.” (P13)
Platform concerns	“I think I'm using it just the same in the last years, but I've become more cautious because unfortunately, Twitter is not Twitter anymore. It is X. And politically, I do not feel aligned with the owner.” (P11)

### Domain 1: Access to Neurology

PIF as a neurologist is fundamentally dependent on access to a community of neurologists. Participants shared how X “opened doors” (P10) for them to become involved in the neurology community beyond initial virtual involvement. Three major themes were identified: participants' reasons for joining, X supporting engagement and opportunities beyond [their] institution, and X as an opportunity for networking.

#### Reasons for Joining

Participants' reasons for joining #NeuroTwitter were diverse. The impact of the COVID-19 (Coronavirus disease of 2019) pandemic and the accompanying “desperation” (P3) played an important role for many. With the cancellation of several in-person opportunities, X emerged as a free and accessible alternative to navigate virtual interviews and find research opportunities. Even when not directly motivated by the pandemic, superusers often referred to their desire to access resources or keep in touch with other neurologists as key motivating factors.

#### Beyond Their Institution

Many users noted staying active on X because their engagement permitted access to resources, mentors, and opportunities beyond their institution. Many participants (61.11%) were IMG trained and looking for opportunities to connect with the US-based Neurology community; others were from medical schools that did not have a neurology clerkship. For these individuals, X emerged as an important means to access neurologists and attain resources or experiences that would not have traditionally been available.

#### Opportunities for Networking

Many described how the informal online environment on X dismantled the traditional hierarchy of medicine. Trainees felt empowered to reach out to program directors or well-known neurology educators, and those individuals were often open to connecting, even without a prior relationship. As such, superusers gained unprecedented access to near-peers, program directors, and leaders in the neurology community.

We found that how participants leveraged X depended on their career stage. Many participants were seeking opportunities for the next step in their professional journey. For medical students: learning more about residency programs; for residents: finding mentors, research collaborators, information about fellowships, and mentoring from subspecialist faculty.

### Domain 2: #NeuroTwitter as a CoP

In our study, superusers frequently described how the #NeuroTwitter community had norms of behavior that made connections possible. Users engaged in discussions and shared information and experiences (the Community). This community was sustained by a collective desire for networking, teaching, and learning (the Practice) about Neurology (the Domain). As such, #NeuroTwitter could be conceptualized not just as a digital community, but a CoP.^8^ Major themes in this domain reflect the intersection of the community domain of CoP and PIF including community norms, role models and mentorship, sense of belonging, and engagement.^22^

### Community Norms

Superusers consistently described the #NeuroTwitter community norms as creating a “positive,” (P18) “joyful,” (P2) and “inspiring” (P4) environment. Many described an acute desire to “pay-it-forward” (P11): having received support from the community in their professional development they sought to replicate that for junior members. The openness of the community dismantled traditional hierarchical barriers and allowed robust engagement for many who may otherwise have been excluded from traditional means of PIF.

### Role Models and Mentorship

Because of the informal environment and approachable demeanor of the collective group, superusers frequently described forming connections with and learning from more senior members of the #NeuroTwitter community; many described these relationships in terms of mentoring and role modeling. Superusers also noted how X “democratized” (P15) access to mentorship from neurologists worldwide—role models they would “model their thinking after” (P7), “try to emulate” (P8) or “take cues from what they are doing” (P8).

#### Belonging

These relationships and those formed with near-peers gave superusers a sense of “belonging”—the feeling that they were “part of something” (P6) and had a community that would “offer support” (P13) or “hope during [a] difficult time” (P6).

#### Engagement

Engagement, which can be conceptualized as “the Practice” in this virtual community took on many different forms. Some superusers were content creators (developing posts on original research or creating “tweetorials” on educational topics); other superusers were amplifiers (users that would comment, repost, and share high yield posts). A common element was purposeful use of social media as a professional development tool. Users were careful to deliberately engage only with neurology and neurology-adjacent content, which created tension between their “personal self” and “professional self,” as discussed further in Domain 4.

### Domain 3: Translation of #NeuroTwitter Involvement Into “in Real-Life” Achievements

Professional identity is shaped by becoming competent in a role and by partaking in the symbols and rituals that demonstrate an individual's involvement in the CoP.^5^ For many superusers, active engagement in the #NeuroTwitter community translated into tangible “in real-life” achievements that symbolized their competency and status within the community. This domain encompassed the major themes of acquisition of knowledge, virtual to real-life mentoring, reputation and visibility, and academic achievements. The “in real-life” achievements made possible through deliberate use of social media are depicted in Figure.

**Figure:**
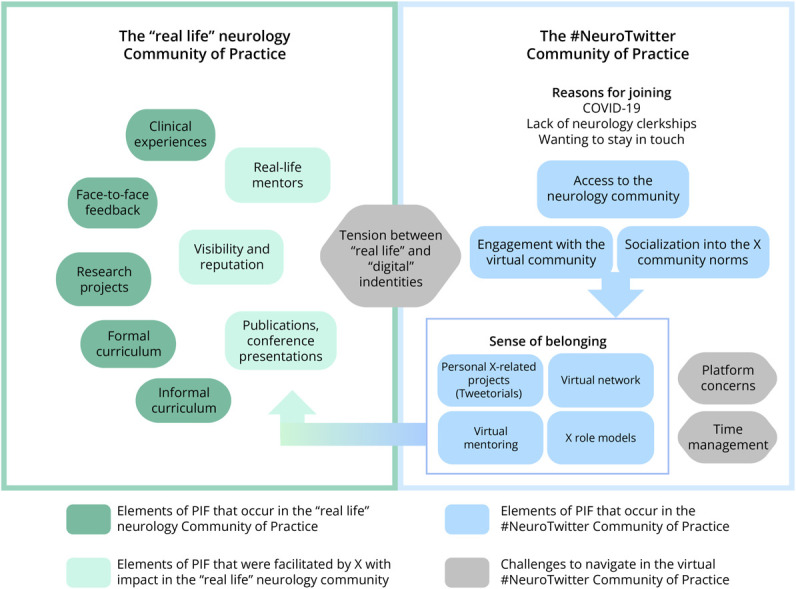
The Relationship Between the #NeuroTwitter Community of Practice and the “Real-Life” Neurology Community of Practice A schematic representation of the intersections between the virtual (#NeuroTwitter) Community of Practice (CoP) and the “Real-Life” Neurology CoP. X superusers joined the virtual CoP for several reasons. Once engaged, active involvement in the digital CoP resulted in virtual mentorship, the identification of role models, and ultimately a sense of belonging in the neurology community. Many of the projects, collaborations, and relationships that were launched or enabled through virtual connections resulted in “Real-Life” achievements and recognition. Accomplishments included research projects, conference presentations, and publications, among others. Challenges to digital engagement included platform concerns, time management, and navigating the tension between digital and real-life identities. Although the virtual #NeuroTwitter CoP is not a substitute for the “Real-Life” Neurology CoP, deliberate use of social media facilitated access to the Neurology CoP and accelerated professional identity formation.

#### Acquisition of Knowledge

Many trainee participants reported they gained knowledge about neurology through X, which translated into improving their diagnostic acumen for their real-life patients. Many trainees noted that teaching and learning was more approachable on X because it was “bite-sized” (P16) and “fun” (P11). Often X was perceived as a faster way to learn, as some participants described how research papers were effectively summarized, and how high-quality educational content was presented in clearer, more accessible ways.

#### Virtual to Real-Life Mentoring

Mentorship is a core component of PIF, as mentors enhance intrinsic motivation and guide trainee's priorities and opportunities.^22^ Often, superusers described how they first approached a mentor through X, such as “my first research mentor, I got through a resident that I reached out to on Twitter” (P5). While some mentoring occurred in the virtual space, the largest benefits were accrued by those whose X mentor became an “in real-life” mentor. Some of these relationships were durable, impactful, and crucial in facilitating residency interviews, research roles and publications, project collaboration, and invitations to conferences.

#### Reputation and Visibility

Even if not facilitated by a mentoring relationship, prominence on X in and of itself enabled superusers to develop visibility and reputation. This was perceived to have tangible benefits during residency or fellowship interviews (Table 2 and eTable 1, available in the Supplement Materials). Visibility also allowed some superusers to create and grow their own projects; many noting that personal visibility often led to project success. Thus, the virtual community not only informed PIF but also enabled the traditional means by which PIF is established “in real life.” Often, the purposeful use of X contributed to opportunities to achieve traditional makers of academic success such as attention to abstracts or publications, which increased opportunities for future collaboration. Participants noted receiving invitations to speak at conferences either about their knowledge of social media or because someone saw their research on X.

### Domain 4: Challenges in Navigating the Digital Environment

While participation in the virtual community was largely perceived as beneficial, superusers also described challenges in navigating the digital environment. These included balancing the tension between one's digital, personal, and professional identities, time management, and platform concerns.

#### Tension Between One's Digital, Personal, and Professional Identities

In all PIF, trainees must navigate the tension between their existing personal identities and their professional identities through the complex process of socialization.^5^ In the virtual environment, many trainees described not only having to navigate a tension between their personal and professional identities, but also the challenge that who they were in the virtual space was “a brand” (P8). Another participant summarized this tension between their real and digital identity as:

“If we aren't being our true, authentic selves … it can make it harder to talk about difficult topics in training and being a physician in general. And I think that that is one of the unseen side effects and downfalls of social media is if we aren't truly being authentic, it makes it more difficult to be vulnerable. And then we become isolated in these self-made silos of like, well, I'm going through this, but it seems like everyone else around me is thriving in their professional lives.” (P7).

#### Time Management

Superusers noted a challenge with “time management.” Content creation and amplification required significant time commitment, which they had to balance with patient care, research, and other professional and personal responsibilities.

#### Platform Concerns

A final challenge to consider was how participants navigated the ongoing evolution of the digital platform. Superusers mentioned distrust of the platform since it had changed, with a trend toward negative attitudes or concerns about the platform.

## Discussion

In a qualitative semi-structured interview study with neurology trainee X superusers, we found 4 major domains regarding the role of #NeuroTwitter in trainee PIF: access to neurology, #NeuroTwitter as a CoP, translation of #NeuroTwitter involvement into “in real-life” achievements, and challenges in navigating the digital environment. Our study offers insights into the perspectives and experiences of X superusers on the role of social media engagement in PIF in neurology, highlighting how purposeful engagement with social media mirrored and, in some cases, accelerated “in real-life” PIF (Figure). Furthermore, our study underscores how a digital community may operate as a parallel CoP and promote socialization into the neurology profession. Although participation was largely described in positive terms, trainees navigated several challenges in the digital environment including the tension between their “real” and “digital” persona, time management, and changes in the platform.^14^

In prior work published during the period when our superusers were most active on X (circa 2020–2023), social media users described participation on X or Twitter (as it was known before late 2023) as being “In the Digital Room Where It Happens” or as “The Network that Never Sleeps” in which there is a multinational community engaged in continual real-time learning free of the traditional barriers that may limit access.^23,24^ Savvy social media users, like those in our sample, often came to X specifically to pursue a professional goal, describing a lack of resources or opportunities at their home institution as the reason why they “ended up looking to social media to help build these connections and find mentors that weren't at [my] home institution” (P7). Many described how X not only democratized access to knowledge but also access to institutions, program directors, mentors, research collaborators, and role models. It allowed users who might otherwise have been excluded from traditional pathways of networking to “get their foot in the door” (P7). The experience of these superusers highlights the growing importance of virtual platforms to promote access for individuals who have been historically excluded from traditional means of networking and professional socialization. A study from the era of virtual interviewing and in-person “second looks” highlighted how those who could not attend in-person second looks often were hampered by a lack of time and insufficient funds.^25,26^ These concerns were mirrored in the language of our participants, who noted that X was often a substitute for the in-person experience they could not have due to international status, travel expenses, or COVID-19 closures. The success of superusers to translate their virtual experiences into real-life achievements emphasizes how the virtual space may democratize access and gives credence to the need to explore other ways in which traditional learning can be provided more equitably such as through virtual rotations,^27^ online curricula,^28^ open-access education.^29^

Superusers' experiences also shed light on how the #NeuroTwitter community can be conceptualized as a CoP, comprised of individuals with shared interests in the practice of neurology. Superusers often first joined X to gain neurology knowledge, appreciating the opportunity for open-access education and tailored, fast-paced teaching. They acquired neurology knowledge but also the social norms of the community. Many appreciated the openness of role models and the willingness of virtual mentors to directly engage. Many noted that because they received support, they wanted to “pay it forward” (P13), which continued to create a sense of inclusion and community. Some went on to promote the content of others or develop their own. This is an important transition, as Creuss et al. emphasized the importance of social learning theory in PIF—that learning among junior members is “situated” within a shared domain (i.e., Neurology) and that to grow in one's professional identity, one must adhere to the norms of the community, cultivate a sense of belonging to that group, and enact the practices of the profession.^5^ By feeling part of the community and developing a strong sense of belonging, superusers began enacting the “practice” of this community—developing education content, participating in collaborative research, or securing mentorship. In doing so, they moved from the periphery towards the center of the CoP and became involved in many activities valued in the “real-life” Neurology CoP. Thus, our participants demonstrated being able to leverage the virtual CoP to promote academic development and scholarship, providing empiric evidence for a theoretical benefit ascribed to virtual CoPs.^30^

For many superusers, mentorship emerged as the most transformative outcome of digital participation. Our participants shared mentors were easier to reach on X, and finding mentors led to tangible real-world benefits, blurring the lines between trainees' social media and in person network, emphasizing the value of mentorship in medical education, and further underscoring the importance of the virtual community to promote equity.^7,22,30,31^

However, despite participants often noting the benefits of participation on X, an important challenge for trainees was navigating the tension between their professional, digital, and “real” self. This experience mirrors the tension many medical trainees face as they navigate PIF and professional development in the medical CoP in real life.^32,33^ However, there is perhaps even more complexity in the virtual space as users often must deliberately craft various aspects of their personal and profession identities and respond to community clues of how they should behave.^14^ This has been described as online impression management, a process in which social media users display certain aspects of themselves based on their perception of who is watching, often at the expense of authenticity.^34^ Furthermore, there are potentially higher costs for professionalism slips online, given the public nature of these forums; an error can compromise or irrevocably damage professional reputation.^35^ Navigating the tensions in crafted identities can interfere with how medical students interact with one other, with their attendings, and with patients, and add tension and uncertainty to their daily lives.

In addition to navigating dissonance between their real and digital identities, participants consistently echoed that deliberate use of X was a time-intensive process. Some described this as contributing to “burn out” (P14), being “addictive” (P13), and taking time away from other hobbies or work. Medical trainees are already spending less time at the bedside than in previous eras; recent observational studies estimate medical interns spend only 13.4% of daily work time in patient rooms.^36^ Although our study did not quantify the amount of time superusers were active on the platform, time spend active on social media was likely at the expense of other activities including in-person PIF opportunities. If overused, involvement in the virtual environment could play a role in increasing errors and burnout as well as weakening the doctor-patient relationship.^37^ Fortunately, many of our participants had insight to these challenges. Most mentioned actively limiting their contact with X when it conflicted with clinical responsibilities, some described deleting the application altogether when it was felt to be no longer serving them.

#NeuroTwitter can be conceptualized as a digital neurology CoP, and undoubtably played an important role in the PIF of many the most invested users among our cohort. However, isolated use of the online community cannot replace the personal and professional development that occurs for trainees entering a profession in real life. For example, role-modeling, mentorship, and experiential learning all play an incredibly important role in PIF. To be maximally effective however, these processes require guided reflection and feedback. This may be challenging to accomplish in the social media space, as trainees do not receive feedback on their doctoring, bedside manner, or other core skills for PIF in medicine in a virtual community. Moreover, digital communities may not deliver challenging but honest feedback about what needs to be improved (e.g., such as the approach to a difficult case). Although what “happens” in the digital space is important for personal and professional development, it should be viewed as a complement to rather than a replacement of the PIF occurring in real life.

Despite X's important role in leveling the playing field and enhancing access to neurology for a diverse group of trainees, the online neurology community is shrinking. In our study, many superusers noted that they recently stopped using X or were not engaging with the platform as frequently. Using Symplur analytics, over a month-long period in August 2025, there were 505 posts with the hashtag #NeuroTwitter—if scaled, this estimates roughly 6,000 posts annually. This is orders of magnitude below the 210,000 #NeuroTwitter posts reported by Symplur in 2022–2023. The dwindling of the #NeuroTwitter community specifically has been described in a recent editorial.^38^ However, what may have been underappreciated—and which is substantiated by our research—is that the slow dismantling of this community may have a disproportionate impact on those who do not otherwise have the means to access traditional forums for networking and mentoring, such as IMGs and students from medical schools without a neurology clerkship. The erosion of the #NeuroTwitter community leaves a concerning void, and although there are burgeoning neurology-focused communities on BlueSky, TikTok, Instagram, and LinkedIn, it is unclear which of these platforms will ultimately fill that void. Our research identifies not a single platform, but rather potentially transferrable qualities of a platform that may be important for supporting trainees' success: democratizing access, role modelling, generosity, optimism, and connection, creating opportunities for mentorship and collaboration, and supporting a spirit of giving back to promote mutual community benefits.

Limitations of our study include initial purposive sampling among superusers known to the researchers. However, we used a criterion method to mitigate bias by establishing a priori criteria for X “superusers” that had to be met by all participants and supplemented initial sampling with snowball sampling to broaden the diversity of participants. Purposeful criterion and snowball sampling may have led to a selection bias in participants which may limit transferability. Reaching thematic saturation helped ensure the trustworthiness of the data. Our participants may have been subject to recall bias. We note that many participants were IMGs, which represent an outsized sample compared to the broader current neurology resident population in the United States. We only analyzed 1 platform (X), and as many of our participants noted, this platform has changed in the last several years. Our findings may not be transferrable to other social media platforms nor may they be transferable to other learner groups or medical specialties. We specifically focused on superusers; whether and how their experiences compare with the more casual user needs to be further explored.

The #NeuroTwitter community can be conceived as a CoP in which community members interacted through shared interests, practice, and learning. Purposeful and consistent engagement in the neurology social media community on X supported PIF among neurology trainees, paralleling processes of PIF which occur in real life. As the neurology community inevitably shifts away from one social media platform to others, our study offers potential lessons for how these platforms may positively support and enable trainees' PIF in digital spaces.

## Glossary

CoP: community of practice
PIF: professional identity formation
